# Journal of health, population and nutrition: celebrating an exciting new phase

**DOI:** 10.1186/s41043-015-0024-y

**Published:** 2015-08-13

**Authors:** Abdullah H. Baqui

**Affiliations:** Department of International Health Director, International Center for Maternal and Newborn Health Johns Hopkins Bloomberg School of Public Health, Johns Hopkins University, Room E-8153, 615 N Wolfe Street, Baltimore, MD 21205 USA

We are pleased to announce a new phase of the *Journal of Health, Population and Nutrition* (JHPN) into an open access, peer-reviewed online journal under the sponsorship of BioMed Central. While celebrating the start of an exciting new phase, it seems appropriate to reflect on the journal’s impressive history and to present some initial plans for the future of this new phase of the journal. *Journal of Health, Population and Nutrition* (JHPN) indeed has a rich and illustrious history. The International Centre for Diarrhoeal Diseases Research, Bangladesh (icddr,b), a world‐class public health research organization in a developing country, first launched the *Journal of Diarrhoeal Diseases Research* in 1983 and the journal took its current name in 2000. During the period 2000–2015, under the management of icddr,b, and with an eminent international editorial board, the journal became internationally renowned for its rigorous standards and its special focus on research of relevance to developing countries.

The new incarnation of the journal will keep this special focus on the developing world. Under BioMed Central, we aim to offer an open access forum for scientifically sound and innovative articles across the broad areas of health, population and nutrition. Topic areas will include traditional areas of global health, such as maternal, newborn and child health, under-nutrition, common illnesses and injuries and determinants of population health. Global burden of disease studies demonstrate that the epidemiological transition is already well advanced and the poorest in developing countries face a triple burden that includes communicable diseases, non-communicable diseases and illnesses linked to socio-behavioural factors. The facts suggest that public health research, policy, and programs in resource poor countries need to adjust. JHPN, therefore, is adapting to address this shift in disease burden occurring in developing countries.

Although the journal is entering a new phase, we decided to keep the name to make us mindful of the journal’s rich history. We hope, however, that as open access, international journal with broad focus on health, population and nutrition, streamlined review, and renewed emphasis on the quality and relevance of the papers, we can attract wider readership. With more downloads and more citations, we should be able to earn a higher impact factor. We hope our transformation will contribute to improvements of health, reduction of health disparities, and protection against global threats to health through sharing of innovations and by promoting exchange of information.

The articles will be published as soon as they are accepted and will be archived in BioMed Central which will also allow the access by readership. Authors will continue to hold copyright for their work and will be able grant anyone the right to reproduce and disseminate the article, provided that it is correctly cited and no errors are introduced. We hope that the open access nature of JHPN will promote exchange of ideas internationally.

Manuscripts submitted to JHPN will be reviewed independently by at least two experts in the field. We are interested in publishing articles of the highest quality and originality. Our goal is to make JHPN an important journal by providing opportunities for communication among the scientists in the field of health, population and nutrition. We look forward to your submission.

